# Changes in Mortality Related to Traumatic Brain Injuries in the Seychelles from 1989 to 2018

**DOI:** 10.3389/fneur.2021.720434

**Published:** 2021-08-27

**Authors:** Anne Abio, Pascal Bovet, Bernard Valentin, Till Bärnighausen, Masood Ali Shaikh, Jussi P. Posti, Michael Lowery Wilson

**Affiliations:** ^1^Injury Epidemiology and Prevention Research Group, Turku Brain Injury Centre, Division of Clinical Neurosciences, Turku University Hospital and University of Turku, Turku, Finland; ^2^Heidelberg Institute of Global Health, University of Heidelberg, Heidelberg, Germany; ^3^University Center for Primary Care and Public Health (Unisanté), Lausanne, Switzerland; ^4^Ministry of Health, Victoria, Seychelles; ^5^Department of Neurosurgery and Turku Brain Injury Centre, Neurocentre, Turku University Hospital and University of Turku, Turku, Finland

**Keywords:** traumatic brain injuries, Seychelles, mortality, low-middle income country, Africa

## Abstract

**Introduction:** Traumatic Brain Injuries (TBIs) are a significant source of disability and mortality, which disproportionately affect low- and middle-income countries. The Republic of Seychelles is a country in the African region that has experienced rapid socio-economic development and one in which all deaths and the age distribution of the population have been enumerated for the past few decades. The aim of this study was to investigate TBI-related mortality changes in the Republic of Seychelles during 1989–2018.

**Methods:** All TBI-related deaths were ascertained using the national Civil Registration and Vital Statistics System. Age- and sex-standardised mortality rates (per 100,000 person-years) were standardised to the age distribution of the World Health Organisation standard population.

**Results:** The 30-year age-standardised TBI-related mortality rates were 22.6 (95% CI 19.9, 25.2) in males and 4.0 (95% CI 2.9, 5.1) in females. Road traffic collisions were the leading contributor to TBI-related mortality [10.0 (95% CI 8.2, 11.8) in males and 2.7 (95% CI 1.8, 3.6) in females, *P* > 0.05]. TBI-related mortality was most frequent at age 20–39 years in males (8.0) and at age 0–19 in females (1.4). Comparing 2004–2018 vs. 1989–2003, the age-standardised mortality rates changed in males/females by −20%/−11% (all cause mortality), −24%/+39.4% (TBIs) and +1%/+34.8% (road traffic injury-related TBI).

**Conclusion:** TBI-related mortality rates were much higher in males but decreased over time. Road traffic collisions were the single greatest contributor to TBI mortality, emphasising the importance of road safety measures.

## Introduction

Traumatic brain injuries (TBIs) are a major cause of mortality and disability worldwide (1–3). TBIs are mainly caused by external kinetic forces to the head—which occur frequently in the context of road traffic collisions, interpersonal violence, subsequent to falls and during sporting activities (4–7). Globally, it is estimated that approximately 27–69.0 million out of 7.3 billion people are diagnosed with TBIs annually, with an incidence of 351–939 cases per 100,000 population (8, 9). On the other hand, an earlier study from 2007 found the global annual incidence at 106 cases per 100,000 population (10). The increase reflects a rise in the TBI incidence over time. The occurrence of TBIs among the younger population is mainly due to collisions in the road environment, while falls account for a higher proportion of TBIs among older people (1, 2, 11–13). Persons who have been diagnosed with a TBI are often affected by long-term disabilities including cognitive and physical impairments, behavioural changes, impaired attention and psychological problems such as depression (1, 4, 11).

Mortality due to TBIs is considerably higher in low- and middle-income countries (LMICs) (8, 14, 15). The annual incidence of TBI was estimated to be 49.9 million out of 6.1 billion people (0.8%) in LMICs, with 7.9 million out of 990.2 million (0.8%) being in the African region alone, compared to 17.9 million out of 1.1 billion (1.5%) in all High Income Countries (HICs) (8). This could be related to a higher overall frequency of external causes of mortality in LMICs, including higher rates of road traffic injuries as a result of rapidly increased motorisation and limited access to adequate management for TBI patients (5, 14). Unintentional falls among older people have been linked to shortened life expectancy and higher occurrence of comorbidities such as Parkinson's disease, Alzheimer's disease, incontinence, mobility limitations, visual and cognitive impairments and inadequate medication adherence (12, 13, 16–21). Additionally, TBIs represent a large economic burden in terms of health care, loss of productivity and income, both at the individual and national levels (22).

TBI-related mortality rates differ widely between populations. Studies conducted in LMICs have found higher rates of TBI-related mortality in rural vs. urban populations, with evidence of a significant gendered gradient (1, 13, 23, 24). Systemic issues within the healthcare systems of resource-limited settings often pose challenges, including delay in accessing health providers, ill-functioning equipment, lack of diagnostic equipment, and inadequate case management, which can adversely impact on outcomes after TBIs (14).

A limited number of studies on external causes of mortality and TBI have been conducted in LMICs, which could be attributed to a paucity of quality data and other funding priorities (25, 26). Therefore, it is important to conduct nationwide population-based research on TBI mortality in less studied areas. The availability of mortality data from the Civil Registration and Vital Statistics system in Seychelles, covering a span of at least 30 years, provides a valuable data resource for such research. We hypothesise that road traffic crashes are the leading contributor to TBI mortality in Seychelles. The aim of this study was to estimate the changes in TBI-related mortality in the Republic of Seychelles, where the population and cause-specific deaths have been enumerated for several decades and data were available for the period between 1989 and 2018.

## Methods

### Data and Study Population

The Republic of Seychelles is an island archipelago located east of Kenya and north of Mauritius. It is a member of the African Union. The population was 96,762 in 2018, (27) with a majority of inhabitants being of African descent. The population has been enumerated through national censuses every ten years, approximately, for the past few decades, with numbers annually adjusted following census by the administrative authorities (28, 29).

Cause-specific mortality is available from the Seychelles Civil Registration and Vital Statistics system based on death certificates for all deaths in the country (28, 29). Electronic data were available for the period between 1989 and 2018. We previously published trends in mortality from external causes between 1989 and 2018 (28). This study focuses on TBI-related mortality.

The death certificates include 4 fields for underlying causes of death (primary cause, secondary cause, tertiary cause and related conditions). The death certificates are filled out by a licenced physician at the time of death. We considered a TBI to be the cause of death if an entry in a vital statistics record mentioned, in any of these four fields, a skull fracture, intracranial injury, crushing head injury, or an unspecified head injury. If a record in the vital statistics database mentioned a TBI, but other information in the record suggested another primary cause of death (e.g., heart attack, etc.), or if it was not possible to unambiguously determine that the death was actually due to a TBI, the case was not included in the analysis (hence TBI-related mortality rates are likely to be conservative in this study). All TBI-related deaths were individually reviewed by the authors AA and JPP. We used the World Health Organisation International Classification of Diseases, 10th revision (ICD-10) including S06, which corresponds to fractures of the skull, intracranial injuries, crushing injuries of the head, traumatic amputation of part of the head, and other/ unspecified injury of the head.

### Statistical Analysis

The age-standardised mortality rates were directly standardised to the age distribution of the WHO world standard population (30). Age-standardised rates provide frequency estimates that are independent from demographic changes in the interval. Indeed, the population of Seychelles increased substantially in size (from 67,038 in 1989 to 96,772 in 2018) and in age (e.g. 6.4% of the population was aged more than 64 years in 1989 vs. 10.0% in 2018). The age-standardised mortality rates were estimated for two 15-year periods (1989–2003, 2004–2018) to assess changes over time. We also estimated TBI-related mortality rates according to age (0–19, 20–39, 40–59, and 60 years and older), based on all data in 1989–2018. We used 15-year intervals due to the small population and small numbers of TBI-related deaths. Analysis was done using Stata 16 (StataCorp, TX, USA). We tested differences in mortality rates across the two time periods using the Wilcoxon rank-sum non-parametric test (31). Ethical approval and authorisation to use the data were obtained from the Ministry of Health, Republic of Seychelles. Furthermore, the Ethical Commission of the University of Heidelberg, upon assessment, declared that the study did not require additional ethical approval. Analyses were retrospective and involved completely anonymised data.

## Results

Table 1 shows the number of deaths during 1989–2018 related to all causes, all external causes, all TBI-related external causes, as well as the distribution of TBI-related deaths, mechanism of injury and age. During the 30-year interval, TBI-related deaths contributed 277 out of 10,889 deaths (2.55%) in males and 50 out of 8052 deaths (0.62%) in females. TBI-related deaths contributed 278 out of 1,263 deaths (21.9%) due to all external causes in males and 50 out of 349 deaths (14.3%) in females. Hence, the number of TBI-related deaths were much higher in males. The proportions of TBI-related deaths, out of all external causes of death, were also higher in males (21.0%) than in females (14.3%). The TBI-related mortality was highest at the age group 20–39 years in males (39.7%) and at age group 0–19 years (34.0%) in females. The mean age for TBI-related mortality was 38 years [Standard Deviation (SD) = 20] (males: 38, SD 18; females: 36, SD 26) suggesting no difference between sex for the mean age when TBI deaths occurred (*p* = 0.425). Road traffic injuries were the leading cause of TBI-associated deaths (48.0%). Road traffic injuries (males: 125/females: 33), falls (33/2), homicide (16/4) were the three most frequent causes of TBI-related deaths, but external causes of deaths of undetermined intent also contributed substantial numbers (86/11).

**Table 1:** Numbers of deaths due to all causes, all external causes, and external causes related to TBI in 1989–2018.

	**Males (%)**	**Proportion for which TBI was the attributable cause of death out of all external causes**	**Proportion for which TBI was the attributable cause of death out of all deaths**	**Females (%)**	**Proportion for which TBI was the attributable cause of death out of all external causes**	**Proportion for which TBI was the attributable cause of death out of all death**
All causes	10,889 (57.4)			8,072 (42.6)		
All external causes	1,263 (78.4)			349 (21.6)		
All external causes related to TBI	277 (84.7)	21.9	2.5	50 (15.3)	14.3	0.6
*By mechanism of injury*						
Road traffic injuries	125 (79.1)	55.6	1.2	33 (20.9)	57.9	0.4
Falls	33 (94.3)	58.9	0.3	2 (5.7)	25.0	0.02
Homicide	16 (80.0)	18.0	0.2	4 (20.0)	14.8	0.05
Drowning	3 (100.0)	1.0	0.03	0 (0.0)	0.0	0.0
Suicide	2 (100.0)	1.6	0.02	0 (0.0)	0.0	0.0
^*^Other unintentional injuries	12 (100.0)	7.9	0.1	0 (0.0)	0.0	0.0
External causes, undetermined intent	86 (88.7)	50.0	0.8	11 (11.3)	34.4	0.1
*By age group*						
0–19	42 (71.2)	35.0	7.7	17 (28.8)	37.0	4.3
20–39	110 (89.4)	21.7	10.2	13 (10.6)	14.8	3.1
40–59	90 (90.9)	23.7	3.3	9 (9.1)	16.7	0.9
60+	35 (76.1)	13.7	0.5	11 (23.9)	6.8	0.2

* *Other unintentional injuries include hit by an aircraft propeller, rock accidents, epilepsy, helicopter crashes, crushing injuries, boat accidents and falling off a moving vehicle. Percent between brackets shows the percentage from total in males and females*.

**Figure 1:**
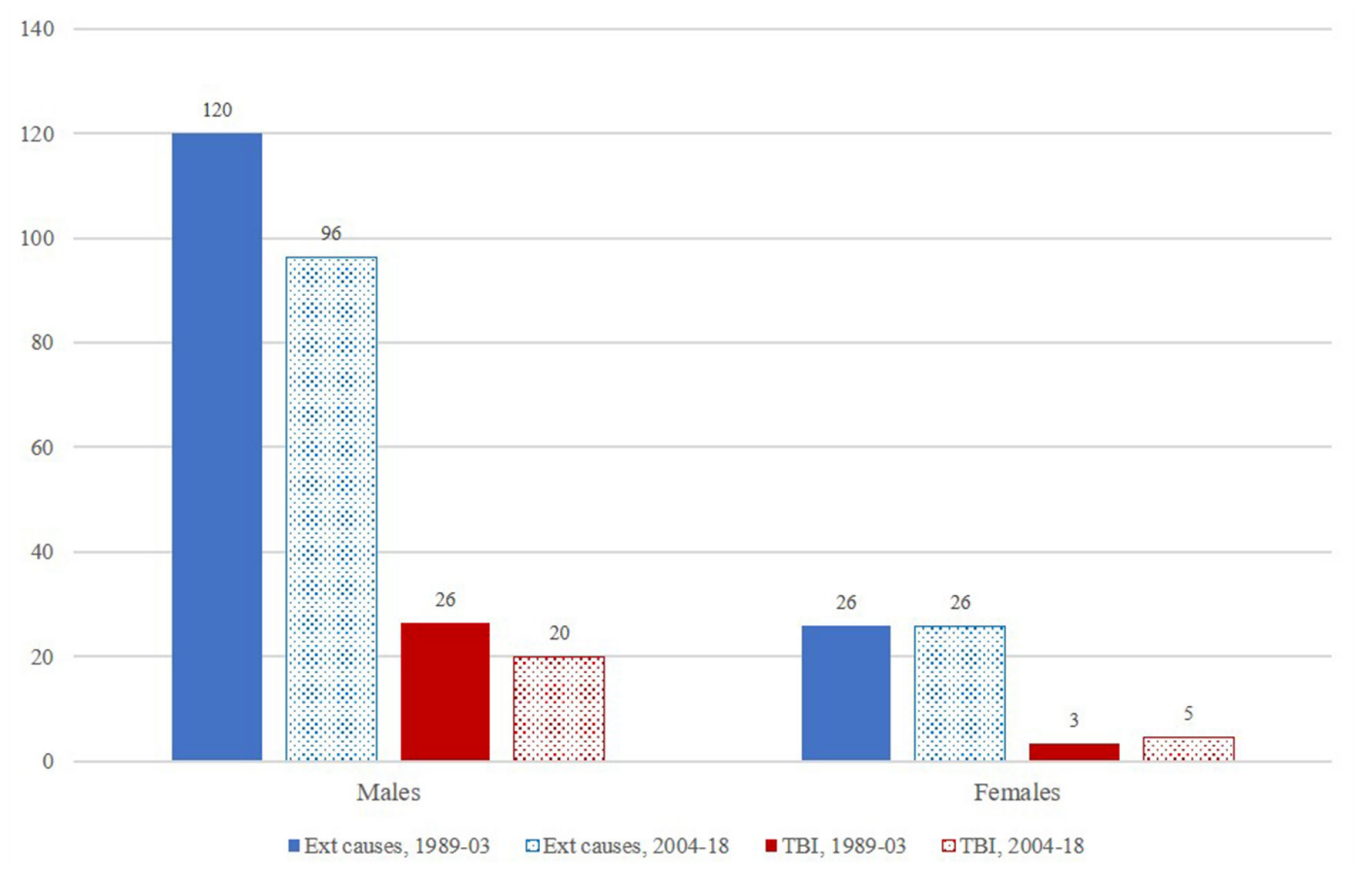
Age-standardised mortality rates for all external causes (including TBIs) and TBI-related deaths only, according to sex and period.

Table 2 shows the age-standardised mortality rates in 1989–2003 and in 2004–2018 for total (all-cause) mortality, all external causes and selected TBI-related causes. In 2004–2018 (“current situation”), the TBI-related mortality rates (per 100,000 persons–years) were 20.0 in males and 4.6 in females. During the 30-year period, from 1989 to 2018, the TBI age-standardised mortality rates were 22.6 (95% CI 19.9, 25.2) among males and 4.0 (95% CI 2.9, 5.1) among females, respectively. Overall, these findings show that the age-standardised TBI-related mortality was approximately 4 times higher in males than in females but the proportions of TBI-related mortality from all external causes of death were similar in males and females (consistent with the nearly 4 times higher age-standardised mortality rates due to all external causes among males) (Figure 1).

**Table 2:** Age-standardised mortality rates for all causes, all external causes and leading specific TBIs during 1989–2003 and 2004–2018.

		**1989–2003**		**2004–2018**			
	**Sex**	**Rate**	**95% CI**	**Rate**	**95% CI**	***p*** **-value**	**RD**
Total mortality (all causes)	M	1,246.5	1,212.7, 1,280.4	997.8	972.8, 1,022.8	<0.001	−20.0
	F	585.4	566.1, 604.8	520.0	504.7, 535.3	<0.001	−11.2
All external causes	M	120.0	110.2, 129.9	96.3	88.8, 103.8	0.125	−19.8
	F	25.8	21.6, 30.0	25.8	22.1, 24.9	0.504	0.0
All TBIs (among all external causes)	M	26.3	21.8, 30.9	20.0	16.7, 23.4	0.247	−24.0
	F	3.3	1.8, 4.7	4.6	3.0, 6.3	0.338	+39.4
Road traffic injury TBIs	M	9.9	7.3, 12.6	10.0	7.6, 12.4	0.781	+1.0
	F	2.3	1.0, 3.6	3.1	1.7, 4.4	0.537	+34.8
Fall-related TBIs	M	1.7	0.5, 2.9	3.5	2.1, 4.9	0.012	+105.9
	F	0.2	<0.1, 0.5	0.1	<0.1, 0.4	0.881	−50.0
Homicide-related TBIs	M	1.4	0.3, 2.5	1.2	0.4, 2.0	0.855	−14.3
	F	0.1	<0.1, 0.4	0.5	<0.1, 1.1	0.428	+400.0
^*^Other TBI-related causes	M	2.0	0.8, 3.2	0.8	0.2, 1.5	0.122	−60.0
	F	0.0	0.0, 0.0	0.0	0.0, 0.0	0.0	0.0
Undetermined intent, external causes of TBI	M	11.2	8.2, 14.3	4.4	2.8, 6.0	0.001	−60.7
	F	0.7	<0.1, 1.3	0.9	0.2, 1.6	0.577	+28.6

* *Others: TBI-related mortality due to drowning, suicide and other unintentional injuries*.

During the period 2004–2018, the main causes for TBI-related mortality were road traffic injuries (males: 10.0; females: 3.1), fall-related TBIs (3.5; 0.1), and homicide-related TBIs (1.2; 0.5), with substantial TBI-related mortality from undetermined causes in males (males 4.4; females 0.9). Road traffic injuries corresponded to 50% (10.0/20.0) of the overall TBI mortality in males and 67% (3.1/4.6) in females. Considering the period 1989–2018 (30-years), the age-standardised TBI mortality from road traffic injuries was 10.0 (95% CI 8.2, 11.8) in males and 2.7 (95% CI 1.8, 3.6) in females. These findings emphasise that road traffic injuries are the leading contributor to TBI-related mortality in both males and females.

Comparing data in 1989–2003 vs. 2004–2018, Table 2 shows that TBI-related age-adjusted mortality rates decreased by 24.0% (*p* > 0.05) in males. Among females, TBI-related mortality rates increased by 39.4% (*p* > 0.05, admittedly based on very small numbers). Although the small mortality rates for cause-specific TBIs (particularly among females) prevent drawing solid conclusions, it can be noted that mortality rates from road traffic collisions slightly increased, although it was not statistically significant.

Table 3 shows the age-adjusted mortality rates by age group. Mortality rates for TBI were highest in males aged 20–39 years (8.0) and at ages 40–59 (7.4), and in females aged 0–19 years (1.4). In contrast, the lowest mortality rates for TBI mortality, were 3.4 at age 0–19 for males and 0.8 at age 40–59 for females.

**Table 3:** Age-standardised TBI-related mortality rates according to period, sex and age.

	**Males**				**Females**			
	**1989–2003**	**95% CI**	**2004–2018**	**95% CI**	**1989–2003**	**95% CI**	**2004–2018**	**95% CI**
All TBIs								
0–19	3.7	2.2, 5.2	3.1	1.7, 4.5	1.4	0.5, 2.4	1.4	0.4, 2.4
20–39	9.0	6.7, 11.3	7.1	5.2, 9.1	0.6	<0.1, 1.2	1.4	0.5, 2.3
40–59	8.4	5.6, 11.1	6.9	5.0, 8.7	0.7	<0.1, 1.6	0.8	0.2, 1.4
60+	5.0	2.8, 7.3	2.8	2.8, 7.3	0.5	<0.1, 1.1	1.0	0.3, 1.8
Road traffic injury with TBI								
0–19	2.8	1.5, 4.1	2.4	1.2, 3.7	1.0	0.2, 1.7	0.9	0.1, 1.7
20–39	4.0	2.5, 5.6	4.4	2.9, 5.9	0.5	<0.1, 1.0	1.1	0.3, 1.9
40–59	1.9	0.6, 3.2	2.4	1.3, 3.5	0.7	<0.1, 1.6	0.5	<0.1, 1.0
60+	1.1	<0.1, 2.1	0.7	<0.1, 1.4	0.2	<0.1, 0.5	0.5	<0.1, 1.0
Falls with TBI								
0–19	0.2	<0.1, 0.5	0.3	<0.1, 0.8	0.2	<0.1, 0.5	0.0	0.0, 0.0
20–39	0.3	<0.1, 0.7	1.0	0.2, 1.7	0.0	0.0, 0.0	0.2	<0.1, 0.4
40–59	0.7	<0.1, 1.5	1.5	0.7, 2.4	0.0	0.0, 0.0	0.0	0.0, 0.0
60+	0.5	<0.1, 1.3	0.7	<0.1, 1.4	0.0	0.0, 0.0	0.0	0.0, 0.0
Homicide with TBI								
0–19	0.0	0.0, 0.0	0.0	0.0, 0.0	0.0	0.0, 0.0	0.2	<0.1, 0.5
20–39	0.5	<0.1, 1.0	0.5	<0.1, 1.1	0.0	0.0, 0.0	0.2	<0.1, 0.4
40–59	0.5	<0.1, 1.1	0.5	<0.1, 1.0	0.0	0.0, 0.0	0.1	<0.1, 0.4
60+	0.5	<0.1, 1.3	0.2	<0.1, 0.5	0.2	<0.1, 0.5	0.0	0.0, 0.0
^*^Others with TBI								
0–19	0.2	<0.1, 0.5	0.2	<0.1, 0.5	0.0	0.0, 0.0	0.0	0.0, 0.0
20–39	1.1	0.3, 1.9	0.3	<0.1, 0.7	0.0	0.0, 0.0	0.0	0.0, 0.0
40–59	0.2	<0.1, 0.7	0.4	<0.1, 0.8	0.0	0.0, 0.0	0.0	0.0, 0.0
60+	0.5	<0.1, 1.3	0.0	0.0, 0.0	0.0	0.0, 0.0	0.0	0.0, 0.0
Undetermined intent with TBIs								
0–19	0.6	<0.1, 1.2	0.2	<0.1, 0.5	0.3	<0.1, 0.8	0.4	<0.4, 0.9
20–39	3.1	1.7, 4.4	1.0	0.2, 1.7	0.2	<0.1, 0.5	0.0	0.0, 0.0
40–59	5.1	3.0, 7.3	2.0	1.0, 3.0	0.0	0.0, 0.0	0.1	<0.1, 0.4
60+	2.4	0.8, 3.9	1.2	0.3, 2.1	0.2	<0.1, 0.5	0.5	<0.1, 1.0

* *The category “Others” includes TBI-related mortality due to drowning, suicide and other unintentional injuries*.

## Discussion

During the three decades studied, we found that TBI-related mortality in the Republic of Seychelles was approximately 4–5 times higher among males and highest at age 20–39 in males and at age 0–19 in females. Comparing age-standardised rates in 1989–2003 vs. 2004–2018, TBI-related mortality decreased in males but did not change in females. Road traffic injuries were the leading contributor to TBI-related mortality.

By international comparison, the mortality rates associated with TBI in the Seychelles were similar to those reported in China among males, but were lower among females (1). Both studies used a civil registration and vital statistics system that was representative of the entire country. However, the TBI-related mortality among males was higher in Seychelles than reported in studies from Austria and Ecuador, but lower than in Finland and in the USA (24, 32–35). The fairly high rates in Seychelles may be partly attributed to the high road traffic injury mortality rates, consistent with high rates in LMICs but was also lower (17.5%) than the global average of 23% (28, 36). The lower rates in Seychelles compared to Finland (22.6 vs. 34.8 per 100,000) may also be attributed to the inclusion of the paediatric population in the former but not in the latter (32). However, other studies in Finland found lower rates (17–18 per 100,000) (33, 37). Few countries from the African region have civil registration systems that cover their respective entire population. Studies from the region have mostly focused on hospital records which may not capture deaths occurring outside of clinical settings (5–7, 38, 39).

The TBI-related mortality was highest at age 20–39 years in males but at age 0–19 years in females in Seychelles. In other countries, the mortality rate was highest above the age of 50 years in Rwanda (in both males and females) (25); and above the age of 60 years in Ethiopia (38). However, direct comparison is difficult as mortality in these other countries was based on hospital admissions, which may not represent the situation in the whole population. The study conducted in China reported that the age-standardised TBI-related mortality rates were highest among persons 75 years or older (1). Obviously, the peak age for TBI-related mortality rates will depend on the predominant underlying causes (e.g. road traffic collisions or homicide predominately occurring in young adults vs. falls at older ages).

Road traffic crashes were the leading mechanism of TBIs regardless of sex in Seychelles; however, the vital statistics do not generally include information on whether a deceased person was a vehicle driver or passenger or a pedestrian. Similarly, road traffic injuries were the leading cause of TBI-related mortality in various studies in LMICs (6, 7, 25, 38). The age-standardised mortality from TBIs related to road traffic injuries was similar in Seychelles and in China, where the rates ranged from 7.7 to 12.4 among males and 2.5–4.1 among females per 100,000 person-years (1).

Falls were the second specific cause of TBI-related mortality in Seychelles, consistent with findings in many other countries, particularly HICs that have high proportions of older persons (1, 6, 21, 38, 40–42). In Seychelles, only 17% of falls contributing to TBI-related mortality were aged 60 years or older and as many as 42.9% were aged 40–59-years. It must be noted that not all deaths resulting from falls are associated with TBIs, and death can result from various other conditions, e.g. complications after orthopaedic surgery, pulmonary embolism, or infections (16, 43, 44). In this study, none of the deaths from TBI-related falls occurred among females older than 40 years.

Homicide was the third most frequent specific cause for TBI-related mortality. Homicide is not particularly frequent in Seychelles. For example, the public are not authorised to own firearms, in contrast to many countries. A study from the USA reported that firearm injuries were the leading cause of TBI injuries (34). Homicide can be related to stabs, which are rarely directed at the face and do not result in TBI-related death. Other studies from Ethiopia, Malawi, Rwanda, Tanzania, and Uganda have reported that assault (5–7, 25, 38, 39) was the second leading cause of TBI-related mortality after road traffic injuries.

A substantial proportion of TBI-related mortality was due to external causes with undetermined intent which reduced over time. Death certificates, which must be completed by a licenced physician in Seychelles, may not be explicit about the circumstances of death (as opposed to police registries). It is therefore not unexpected that a substantial proportion of TBI-related deaths fall under “undetermined intent”. Further information about TBI-related deaths would be obtained more precisely from police records. The reduction in the TBI mortality cases between the two time periods could be attributed to improved documentation in the death certificates over time (28).

Regarding changes over time (1989–2003 vs. 2004–2018), the TBI-related mortality decreased among males, similarly to the decrease in all-cause mortality and in mortality from all external causes. This may be related to better health conditions and health care over time, as well as the implementation of safety-related policies and programs in many sectors. The TBI-related mortality did not decrease among females, but rates were extremely low in the first place.

Importantly, the TBI-related mortality due to road traffic collisions did not decrease despite more stringent preventive measures relating to traffic control over time. This can partly be explained by the very large increase in cars (an increase by 5–10 times in 30 years) and subsequent increasing traffic density (28), in a context of topographic constraints (steep hills, high density of houses) that limit efforts to further widen roads or make them safer. Traffic injury collisions remain a main area for action to reduce TBI-related deaths.

## Strengths and Limitations

The study has several strengths. First, the population was regularly enumerated and all deaths occurring in the whole population were recorded during the entire study period. Second, data extraction and coding were done in the same manner for all deaths, which minimised errors due to different interpretation of data entries. Thus, the data from 1989 was coded using the ICD codes as soon as it was made available to the researchers. Third, data used in this study included all deaths (numerator) and the entire population (denominator), which ensured that estimates are not only population-based, but represent the exact situation at the whole country level (i.e. not a subsample of the population). Additionally, the causes of death related to TBIs were also reviewed by an experienced neurotraumatologist/neurosurgeon (JPP), which strengthens case validity. There are also several limitations. First, the causes of death entered into the vital statistics database rely on the physicians, often general practitioners, who complete the death certificates. As in many other countries, physicians are often not formally trained on how to complete death certificates along the WHO disease classification procedures. Second, the date of injury was rarely known, and the duration until death could not be assessed, which can complicate the determination of the cause of death, particularly in relation to TBI-related mortality. Third, the number of TBI deaths particularly among females were small which may have affected the stability of female mortality rates. For example, there were 50 female TBI related deaths during the 30-year period. However pooling data across several years helped overcome this accuracy issue. Fourth, the accuracy of data appearing in the death certificates may not be sufficiently detailed to reliably assess the aetiology of all TBIs. Based on this limitation, it was not possible to reclassify the undetermined intent categories to estimate the possibility and/or degree of unmeasured classification bias nor conduct a sensitivity analysis on this sample. As we included only TBIs for which the identification of TBIs as a cause of death was fairly clear on the death certificate, our TBI-related mortality estimates are likely to be conservative (i.e., possibly underestimated).

## Conclusion

The TBI-related mortality was much higher in males than in females, consistent with a similar sex-difference in mortality rates from all external causes. Fortunately, the age-standardised TBI-related mortality rates decreased over time among males. Road traffic injuries continue to be the leading contributor of TBI-related mortality, emphasising the important role of road safety.

## Data Availability Statement

The data is available on request from the Ministry of Health, Seychelles. Requests to access these datasets should be directed to customerservice@health.gov.sc.

## Ethics Statement

Ethical review and approval was not required for the study on human participants in accordance with the local legislation and institutional requirements. Written informed consent from the participants' legal guardian/next of kin was not required to participate in this study in accordance with the national legislation and the institutional requirements.

## Author Contributions

AA led the analysis and the drafting of the paper under the supervision of ML and JP. PB advised in the data analysis and contributed to the writing of the paper. MS, BV, and TB critically reviewed the paper. AA and ML had full access to the data and take responsibility for the results. All authors agreed with the final document.

## Conflict of Interest

The authors declare that the research was conducted in the absence of any commercial or financial relationships that could be construed as a potential conflict of interest.

## Publisher's Note

All claims expressed in this article are solely those of the authors and do not necessarily represent those of their affiliated organizations, or those of the publisher, the editors and the reviewers. Any product that may be evaluated in this article, or claim that may be made by its manufacturer, is not guaranteed or endorsed by the publisher.
